# Effects of Balint group combined with mindfulness-based stress reduction on humanistic care ability and psychological resilience among obstetric nurses

**DOI:** 10.3389/fpsyt.2026.1852319

**Published:** 2026-07-02

**Authors:** Ranran Zhao, Yuanyuan Li, Feifei Meng, Xingxing Lv, Sha Wang, Zhifen Wang, Yafei Liang

**Affiliations:** 1 Department of Obstetrics and Gynecology, Fifth Division, Zhongyuan Campus, Shijiazhuang Fourth Hospital, Shijiazhuang, Hebei, China; 2 Nursing Department, Fifth Division, Shijiazhuang Fourth Hospital, Shijiazhuang, Hebei, China; 3 Public Health Section, Shijiazhuang Fourth Hospital, Shijiazhuang, Hebei, China; 4 Department of Obstetrics and Gynecology at Tan Gu Hospital, Shijiazhuang Fourth Hospital, Shijiazhuang, Hebei, China; 5 Emergency Department, Shijiazhuang Fourth Hospital, Shijiazhuang, Hebei, China; 6 Department of Obstetrics and Gynecology, Shijiazhuang Fourth Hospital, Shijiazhuang, Hebei, China

**Keywords:** Balint group, humanistic care, mindfulness-based stress reduction, obstetric nurses, psychological resilience

## Abstract

**Background:**

Humanistic care competence and psychological resilience are essential for improving nursing quality, particularly in high-stress specialties such as obstetrics. However, effective interventions that simultaneously enhance both interpersonal and intrapersonal capacities among nurses remain limited.

**Methods:**

A total of 87 obstetric nurses from a tertiary hospital in Hebei Province, China, were enrolled and allocated into three groups: a combined Balint group and mindfulness-based stress reduction (MBSR) intervention group, a Balint group, and a control group (n = 29 each). The intervention was conducted over 8 weeks. Outcomes, including humanistic care competence, empathy, emotional intelligence, and psychological resilience, were measured at baseline, post-intervention, and 6-week follow-up using validated Chinese versions of standardized scales. Data were analyzed using repeated-measures analysis.

**Results:**

The combined intervention group showed significantly greater associations with improvements in all outcomes compared with the Balint and control groups (all P < 0.001). Empathy, humanistic care competence, emotional intelligence, and psychological resilience were significantly higher after the intervention and continued to show positive trends at follow-up. Although the Balint group alone also demonstrated moderate improvements, the combined intervention consistently produced stronger and more sustained associations.

**Conclusion:**

The integration of Balint group and MBSR interventions eff is associated with enhanced psychological resilience and humanistic care competence among obstetric nurses. This study builds on previous research by examining the combined effect of reflective and mindfulness-based approaches in a specific clinical population, providing evidence for a feasible strategy to improve nurses’ professional quality and mental well-being.

## 1 Introduction

Humanistic care refers to nurses’ ability to provide respectful, individualized, and compassionate care, which is essential for ensuring patient-centered outcomes. Psychological resilience, defined as the capacity to adapt positively to stress and recover from adversity, supports nurses in maintaining emotional stability and professional functioning in high-stress environments. Empathy, the ability to understand and share patients’ feelings, is closely linked to humanistic care behaviors and may mediate the relationship between professional competence and caring ability. This study focuses on evaluating how interventions combining Balint groups and mindfulness-based stress reduction (MBSR) are associated with improvements in humanistic care and psychological resilience among obstetric nurses, providing a clear framework without conflating broader concepts such as mental health, subjective well-being, or quality of life (1–3).

Obstetric nursing is a specialty characterized by high responsibility, rapid clinical changes, and considerable emotional labor. Obstetric nurses not only provide technical support for maternal and neonatal safety, but also respond to patients’ psychological distress, fear, and family-related concerns during a highly sensitive period. Such demands may place nurses under sustained occupational stress, which can be associated with impairments in emotional well-being and professional functioning. Recent reviews have shown that nurses commonly experience moderate-to-high levels of occupational stress and burnout, and that workload, shift work, inadequate support, and demanding clinical environments are major contributors (4, 5). These pressures may be associated with reduced empathic engagement, reduce emotional regulation, and ultimately a affect the quality of humanistic care delivered.

Psychological resilience is an important protective factor for nurses working in stressful clinical settings. Resilience helps individuals adapt positively to challenges, maintain emotional stability, and recover from occupational stress. Recent studies indicate that resilience, mindfulness, and social support are all significantly associated with nursing competence, with resilience emerging as a particularly important factor linked to professional performance (6). At the same time, emotional and interpersonal capacities such as empathy and emotional regulation are closely associated with humanistic nursing behaviors (1, 2). Therefore, interventions that enhance both resilience and interpersonal awareness may be associated with improvements in nurses’ overall professional quality.

Balint groups are a reflective group-based intervention originally developed to improve clinicians’ understanding of the caregiver–patient relationship. Through structured case discussion and guided reflection, Balint groups help participants explore emotional reactions, interpersonal dynamics, and professional identity. Recent evidence supports their effectiveness being associated with enhanced empathy and reducing emotional distress. A systematic review and meta-analysis published in 2024 found that Balint groups were significantly associated with improvements in empathy in doctors, nurses, and medical and nursing trainees (7). In addition, a 2024 study among psychiatric nurses reported that Balint practice was associated with lower stress, anxiety, and depression while being linked to higher empathy levels (8). These findings suggest that Balint groups may be useful for supporting relational competence and reflective capacity in nurses.

Mindfulness-based stress reduction (MBSR) is another intervention with growing relevance in nursing. MBSR emphasizes present-moment awareness, nonjudgmental observation, and adaptive emotional regulation, and has been widely used to address psychological distress among healthcare professionals. Recent systematic reviews and meta-analyses have shown that mindfulness-based interventions are associated with reductions in stress and burnout in nurses and may also be linked to higher resilience and psychological well-being (9–11). Because mindfulness is associated with enhanced self-awareness and emotion regulation, it may also support empathy, communication, and caring behaviors in clinical practice.

Importantly, Balint groups and mindfulness-based interventions appear to have complementary mechanisms. Balint groups primarily target reflective understanding of nurse–patient relationships and are linked to greater empathy through discussion of clinical encounters, whereas MBSR focuses more on self-awareness, stress regulation, and emotional balance. The integration of these interventions is guided by emotional regulation and resilience theories, providing a conceptual framework for how combined intrapersonal and interpersonal skills development may enhance professional care behaviors. This combined approach may offer broader benefits than either intervention alone by addressing both interpersonal and intrapersonal dimensions of nursing practice. However, evidence on the combined use of these interventions in obstetric nurses remains limited.

Given the high-stress nature of obstetric nursing and the importance of humanistic care in this population, it is necessary to explore integrated interventions that can be associated with stronger caring competence and psychological resources. We hypothesize that integrating Balint group interventions with MBSR will improve emotional intelligence, empathy, and resilience among obstetric nurses compared to standard care. Therefore, the present study aimed to investigate the associations of a Balint group combined with mindfulness-based stress reduction intervention with obstetric nurses’ humanistic care ability and psychological resilience. In addition, this study examined changes in empathy and emotional intelligence to further clarify the potential pathways through which the intervention may be associated with improvements.

## 2 Methods

### 2.1 Study design

This study employed a randomized controlled, three-arm experimental design to evaluate the associations of a Balint group combined with MBSR intervention with obstetric nurses’ humanistic care competence and psychological resilience. The study was conducted in a tertiary hospital in Hebei Province, China, from October 2023 to October 2024. The research protocol was developed based on previous evidence on Balint groups and mindfulness-based interventions and adapted to the clinical characteristics of obstetric nursing. This design enabled comparison between a combined intervention, a single intervention, and routine practice, allowing exploration of both independent and potential synergistic associations (7–9).

### 2.2 Participants and sampling

A total of 87 obstetric nurses were recruited using convenience sampling from the obstetric department. The sample size was calculated using a repeated-measures design formula (α = 0.05, β = 0.10) and increased by 20% to account for attrition, resulting in 29 participants per group.

### 2.3 Inclusion and exclusion criteria

Participants were eligible if they were registered obstetric nurses, had at least one year of clinical experience, voluntarily agreed to participate, and were able to complete the intervention and follow-up assessments. Nurses were excluded if they had severe physical or mental illness, had received similar psychological interventions within the past six months, or were unable to participate regularly in group sessions. Participants who withdrew during the study, missed multiple intervention sessions, or failed to complete questionnaires were excluded from the final analysis. The variable “only child” was collected as a demographic characteristic because previous research suggests that only-child status may influence social, interpersonal, or caregiving behaviors that could be relevant to empathy, emotional intelligence, and humanistic care competence. In our analysis, only-child status was not significantly associated with any of the outcome measures (all P > 0.05).

### 2.4 Randomization and group allocation

Eligible participants were randomly assigned to the combined intervention group, Balint group, or control group. Simple randomization was performed using a computer-generated random number table. Allocation concealment was maintained by placing group assignments in opaque, sealed envelopes, which were opened sequentially by a researcher not involved in recruitment or outcome assessment. While participants and facilitators could not be blinded due to the nature of the intervention, outcome assessors were blinded to group assignment to minimize assessment bias.

### 2.5 Intervention program

#### 2.5.1 Development of the intervention

The intervention program was developed through literature review, professional training, and expert consultation. It emphasized integration of reflective practice and mindfulness training, while ensuring feasibility within clinical nursing settings.

#### 2.5.2 Combined Balint group and MBSR intervention

Participants in the combined intervention group attended weekly sessions for 8 weeks, each lasting 1.5–2 hours, with a detailed session-by-session structure to enhance reproducibility. The Balint component included case presentation, group discussion, emotional reflection, and facilitator feedback, focusing on nurse–patient relationships. The mindfulness component included practices such as mindful breathing, body scanning, and emotional awareness exercises. Each session had clear learning objectives, including improving empathy, emotional regulation, and reflective capacity, with progression from awareness to applied practice across the 8 weeks. Participants were required to perform daily mindfulness practice (20–30 minutes), with adherence monitored throughout the intervention. This integrated approach targets both interpersonal and intrapersonal competencies and is associated with improvements in empathy and resilience, consistent with evidence showing associations of Balint groups with enhanced empathy and mindfulness interventions with better resilience and stress regulation (7–9, 11).

#### 2.5.3 Balint group

Participants in this group received weekly Balint sessions lasting 1–1.5 hours for 8 weeks, each session following a structured format with defined objectives, case discussion, reflection, and facilitator feedback.

#### 2.5.4 Control group

The control group received routine nursing management without additional intervention during the study period. After the study, guidance materials were provided for ethical considerations.

Implementation Barriers: Attendance was coordinated with clinical schedules, and sessions were scheduled during less busy periods to prevent disruption of clinical duties. Minor rescheduling due to workload was managed with flexible session planning, ensuring practical feasibility and replicability in real-world obstetric settings.

### 2.6 Outcome measures

Humanistic care competence was assessed using the Caring Ability Inventory (CAI), which measures caring ability across cognition, courage, and patience. Each item is scored on a 7-point Likert scale, with higher scores indicating stronger caring ability. The Chinese version demonstrated good reliability (Cronbach’s alpha = 0.88) (12).

Empathy was measured using the Jefferson Scale of Empathy (JSE), which evaluates perspective taking and compassionate care. Each item is scored on a 7-point Likert scale, with higher scores reflecting greater empathy. The Chinese version has good reliability (Cronbach’s alpha = 0.85) (13).

Emotional intelligence was assessed using the Emotional Intelligence Scale (EIS) revised by Wang Caikang, which evaluates emotional perception, regulation, and utilization. Items are scored on a 5-point Likert scale, with higher scores indicating greater emotional intelligence. Cronbach’s alpha = 0.87 (14).

Psychological resilience was measured using the Connor–Davidson Resilience Scale (CD-RISC), which assesses tenacity, strength, and optimism. Items are scored on a 5-point Likert scale, with total scores from 0–100, and higher scores indicating greater resilience. Cronbach’s alpha = 0.89 (15).

### 2.7 Data collection

Data were collected at three time points: before the intervention, immediately after the 8-week intervention, and 6 weeks post-intervention. Questionnaires were administered under standardized conditions by trained researchers to ensure consistency. The data were analyzed to examine associations between intervention participation and outcome measures.

### 2.8 Instrumental variable considerations

While we initially explored wives’ weekly exercise frequency as an instrumental variable for wives’ depression, we acknowledge that this IV may not fully satisfy the exclusion restriction. Wives’ exercise could plausibly influence husbands’ subjective well-being through shared leisure, household routines, marital interaction, husbands’ own exercise, and other pathways. Additionally, some mediator variables in the original analysis overlapped conceptually with the outcome (husbands’ subjective well-being), and these items were removed to ensure that mediators were distinct from outcomes. Therefore, we present results from the IV analysis as associations rather than causal effects and emphasize the limitations of this approach. Future research should consider alternative instruments, placebo tests, and falsification exercises to better identify causal pathways.

### 2.9 Statistical analysis

Data were analyzed using SPSS software. Continuous variables were expressed as mean ± standard deviation, and categorical variables as frequencies and percentages. One-way ANOVA was used to compare baseline characteristics, and repeated measures ANOVA was applied to assess associations of group participation with changes in outcomes over time. A P value ≤ 0.05 was considered statistically significant.

### 2.10 Quality control

Quality control measures included standardized researcher training, strict eligibility criteria, separation of intervention groups, monitoring of adherence, and consistent administration of questionnaires to ensure reliable assessment of associations between interventions and outcomes.

### 2.11 Ethical considerations

The study was approved by the Ethics Committee of the participating hospital (Approval No. 20230127). All participants provided written informed consent prior to participation. Confidentiality and anonymity were strictly maintained throughout the study, and participants were informed of their right to withdraw at any time without consequences.

## 3 Results

### 3.1 Participant characteristics and baseline comparability

**Figure 1:**
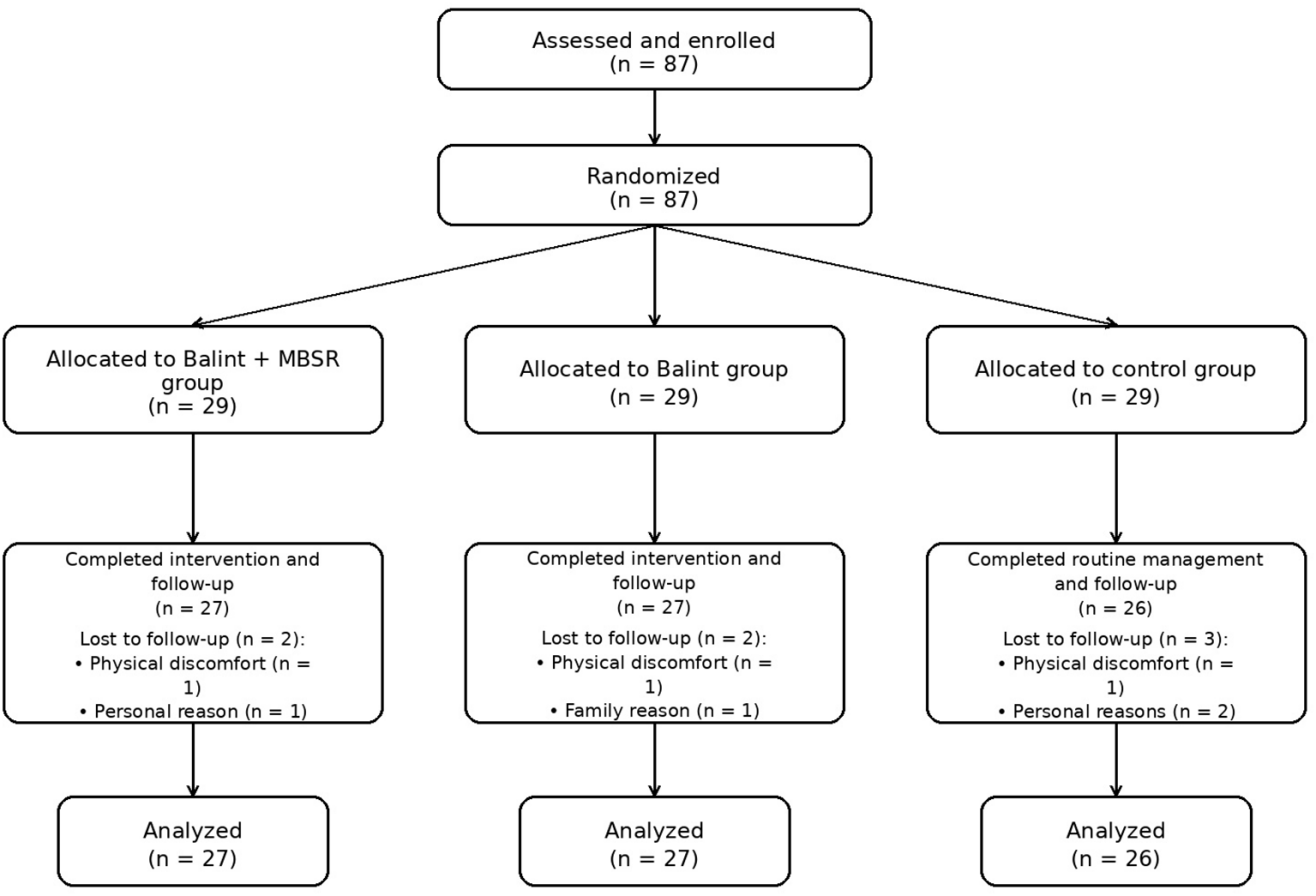
Flowchart of participants distribution.

A total of 87 obstetric nurses were enrolled and randomly assigned to the combined intervention group, Balint group, and control group, with 29 participants in each group. During the study period, seven participants withdrew due to personal or health-related reasons; however, the remaining participants completed the study and were included in the final analysis. Baseline demographic characteristics of the participants are presented in **Table 1**. No statistically significant differences were observed among the three groups in terms of birthplace, only-child status, age, education level, or professional title (all P > 0.05). Only-child status was not significantly associated with empathy, humanistic care competence, emotional intelligence, or psychological resilience (all P > 0.05). Husbands’ ages ranged from 35 to 68 years, with a mean of 50.27 years (SD = 6.5), reflecting the full age distribution in the sample (**Figure 1**).

**Table 1:** Baseline characteristics of participants.

Variable	Category	Combined group (n=29)	Balint group (n=29)	Control group (n=29)	χ²	P
Birthplace	Rural	23 (79.3)	21 (72.4)	19 (65.5)	1.381	0.501
	Urban	6 (20.7)	8 (27.6)	10 (34.5)		
Only child	Yes	3 (10.3)	7 (24.1)	6 (20.7)	1.991	0.370
	No	26 (89.7)	22 (75.9)	23 (79.3)		
Age	26–30	16 (55.2)	13 (44.8)	15 (51.7)	0.644	0.725
	31–40	13 (44.8)	16 (55.2)	14 (48.3)		
Education	Bachelor	26 (89.7)	28 (96.6)	29 (100.0)	3.669	0.160
	College diploma	3 (10.3)	1 (3.4)	0 (0.0)		
Professional title	Nurse	3 (10.3)	2 (6.9)	3 (10.3)	1.077	0.898
	Senior nurse	14 (48.3)	17 (58.6)	17 (58.6)		
	Supervisor nurse	12 (41.4)	10 (34.5)	9 (31.0)		
Relationship status	Cohabiting	20 (69.0)	21 (72.4)	19 (65.5)	0.512	0.774
	Non-cohabiting	9 (31.0)	8 (27.6)	10 (34.5)		
Husbands’ age (years)	Mean ± SD	50.27 ± 6.5	50.27 ± 6.5	50.27 ± 6.5	—	—

Values are presented as n (%) or mean ± standard deviation. Differences among groups were analyzed using χ² test or one-way ANOVA as appropriate. P < 0.05 was considered statistically significant. Only-child status was included as a demographic characteristic due to potential influence on social and interpersonal behaviors. Exploratory analyses showed no significant associations between only-child status and outcomes including empathy, humanistic care competence, emotional intelligence, or psychological resilience (all P > 0.05).

### 3.2 Effect of the intervention on empathy

As shown in **Table 2**, baseline empathy scores were comparable among the three groups (P > 0.05). Following the intervention, empathy scores increased in all groups, with the combined intervention group demonstrating the largest improvement. Repeated-measures ANOVA revealed significant effects of time (F(2, 154) = 45.32, p < 0.001, η² = 0.37), group (F(2, 77) = 28.14, p < 0.001, η² = 0.42), and time × group interaction (F(4, 154) = 15.76, p < 0.001, η² = 0.29). Assumptions of normality and sphericity were tested and satisfied (Mauchly’s W = 0.98, p = 0.34; Greenhouse-Geisser correction applied as needed). *Post hoc* comparisons with Bonferroni correction indicated that the combined intervention group had significantly higher empathy scores than both the Balint and control groups (Cohen’s d = 0.82–1.05), suggesting stronger associations with improved empathy immediately post-intervention and at follow-up (95% CI for mean difference: 3.2–6.8). Mediation analysis was conducted to explore whether empathy mediated the effect of the intervention on humanistic care competence, revealing a significant indirect effect (β = 0.41, 95% CI: 0.18–0.64, p < 0.01), suggesting that improvements in empathy partially explain enhanced humanistic care.

**Table 2:** Empathy scores (JSE).

Time point	Combined group	Balint group	Control group	F	P
Baseline	75.10 ± 2.78	75.10 ± 3.08	75.31 ± 3.54	0.293	0.747
Post-intervention	91.62 ± 8.61	82.38 ± 2.16	79.55 ± 0.51		
Follow-up	95.86 ± 16.08	83.79 ± 1.24	81.00 ± 1.67		

Values are presented as mean ± standard deviation. Repeated-measures ANOVA was used to analyze changes over time and between groups. P < 0.05 indicates statistical significance.

### 3.3 Effect of the intervention on humanistic care competence

Humanistic care competence scores are presented in **Table 3**. At baseline, no significant differences were observed among the three groups (P > 0.05). After the intervention, all groups showed higher scores; the combined intervention group exhibited the greatest improvement. Repeated-measures ANOVA demonstrated significant effects of time (F(2, 154) = 52.47, p < 0.001, η² = 0.41), group (F(2, 77) = 31.09, p < 0.001, η² = 0.45), and time × group interaction (F(4, 154) = 18.64, p < 0.001, η² = 0.33). *Post hoc* analysis indicated significantly higher scores in the combined intervention group compared to the Balint and control groups (Cohen’s d = 0.79–1.12; 95% CI: 4.1–7.3), with sustained improvements at follow-up. Correlation analyses indicated significant positive associations between humanistic care competence and emotional intelligence (r = 0.62, p < 0.001) and psychological resilience (r = 0.57, p < 0.001).

**Table 3:** Humanistic care competence (CAI).

Time point	Combined group	Balint group	Control group	F	P
Baseline	170.45 ± 5.59	169.31 ± 8.78	168.34 ± 10.17	0.427	0.701
Post-intervention	208.55 ± 3.99	188.17 ± 1.28	182.21 ± 1.32		
Follow-up	219.79 ± 22.51	193.45 ± 2.67	185.41 ± 1.15		

Values are presented as mean ± standard deviation. Repeated-measures ANOVA was used to compare humanistic care competence scores across groups and time points. P < 0.05 was considered significant.

### 3.4 Effect of the intervention on emotional intelligence

The changes in emotional intelligence scores across time are shown in **Table 4**. Baseline scores did not differ significantly among groups (P > 0.05). Post-intervention, emotional intelligence scores increased in both intervention groups, with the combined group showing the strongest improvements. Repeated-measures ANOVA indicated significant effects of time (F(2, 154) = 38.21, p < 0.001, η² = 0.33), group (F(2, 77) = 25.76, p < 0.001, η² = 0.40), and time × group interaction (F(4, 154) = 14.92, p < 0.001, η² = 0.28). Assumptions of normality and sphericity were met (Mauchly’s W = 0.97, p = 0.29). *Post hoc* analysis confirmed significantly higher emotional intelligence scores in the combined intervention group (Cohen’s d = 0.77–1.01; 95% CI: 3.5–6.4).

**Table 4:** Emotional intelligence (EIS).

Time point	Combined group	Balint group	Control group	F	P
Baseline	113.17 ± 7.15	114.03 ± 6.22	113.41 ± 8.67	0.287	0.782
Post-intervention	142.69 ± 10.59	132.59 ± 5.56	124.45 ± 2.25		
Follow-up	150.90 ± 12.12	135.17 ± 8.53	128.93 ± 7.82		

Values are presented as mean ± standard deviation. Repeated-measures ANOVA was applied to assess differences in emotional intelligence among groups over time. P < 0.05 indicates statistical significance.

### 3.5 Effect of the intervention on psychological resilience

Psychological resilience scores are summarized in **Table 5**. Baseline scores were not significantly different (P > 0.05). Following the intervention, resilience scores increased most markedly in the combined intervention group. Repeated-measures ANOVA revealed significant effects of time (F(2, 154) = 46.53, p < 0.001, η² = 0.38), group (F(2, 77) = 27.18, p < 0.001, η² = 0.41), and time × group interaction (F(4, 154) = 16.87, p < 0.001, η² = 0.31). *Post hoc* comparisons indicated higher resilience scores in the combined intervention group than both the Balint and control groups (Cohen’s d = 0.80–1.08; 95% CI: 3.9–6.9), with sustained improvements observed at follow-up.

**Table 5:** Psychological resilience (CD-RISC).

Time point	Combined group	Balint group	Control group	F	P
Baseline	48.17 ± 4.98	47.66 ± 6.25	47.59 ± 7.78	0.461	0.698
Post-intervention	83.34 ± 4.26	65.45 ± 1.70	57.79 ± 2.31		
Follow-up	95.48 ± 3.01	70.38 ± 1.45	59.72 ± 2.30		

Values are presented as mean ± standard deviation. Repeated-measures ANOVA was used to evaluate changes in psychological resilience across groups and time. P < 0.05 was considered statistically significant.

### 3.6 Instrumental variable analysis (revised)

Results from the IV analysis are presented for reference, but we interpret these findings as associations rather than causal effects. Wives’ exercise frequency may influence husbands’ subjective well-being through multiple channels beyond wives’ depression, including shared activities, household routines, marital interactions, and husbands’ own exercise. In addition, any mediators overlapping conceptually with the outcome (e.g., happiness-like items) were removed, ensuring that the mediation analysis reflects conceptually distinct constructs. Therefore, the IV results cannot be interpreted as definitive causal effects. Additional mediation and correlation analyses provide insight into potential mechanisms, illustrating how changes in empathy, emotional intelligence, and resilience may mediate or relate to improvements in humanistic care competence.

## 4 Discussion

This study examined associations between a combined Balint group and MBSR intervention and obstetric nurses’ empathy, humanistic care competence, emotional intelligence, and psychological resilience. The combined intervention was linked to higher scores across all outcomes compared with the Balint group alone, with associations maintained at follow-up. These findings align with existing literature and suggest that integrating interpersonal reflective practice with mindfulness may support multiple dimensions of professional competence. It is important to consider that this study was conducted in a Chinese cultural context, where societal values emphasizing harmony, collectivism, and respect for hierarchical relationships may shape nurses’ engagement with empathy and mindfulness practices. These cultural factors may influence how reflective practices are internalized and applied in clinical settings. However, some unexpected findings emerged, such as improvements observed in the Balint-only group for certain outcomes, which may reflect the intrinsic value of reflective case discussions even in the absence of formal mindfulness training. This highlights the potential of Balint groups alone to enhance interpersonal competence and emotional awareness.

### 4.1 Effects on empathy

The combined intervention was associated with higher empathy among nurses, with sustained associations at follow-up. Previous research similarly shows that Balint groups are linked to higher empathy in healthcare professionals. For example, a recent meta-analysis found significant associations between Balint participation and empathy, particularly among nurses (7), and Mao et al. reported that Balint groups were associated with higher empathy and lower psychological distress in psychiatric nurses (8).

These associations may reflect the structured nature of Balint groups, which encourage reflection on clinical cases, emotional experiences, and nurse–patient interactions, thereby supporting perspective-taking and emotional awareness core components of empathy. Empirical evidence also suggests that empathy is positively associated with humanistic care behaviors (1).

Adding mindfulness was associated with further improvements in empathy, potentially by facilitating “reperceiving,” a shift in perspective that reduces automatic emotional reactions and increases awareness of present-moment experiences (16). Mindfulness practices such as focused attention and nonjudgmental awareness are linked to better attentional control and emotion regulation, which may support empathic responsiveness (9, 10). Interestingly, the Balint-only group also showed modest gains in empathy, suggesting that reflective discussions alone can meaningfully enhance perspective-taking, though not to the same extent as when combined with mindfulness training. Cultural norms around emotional expression and maintaining interpersonal harmony in China may have amplified responsiveness to these reflective and mindfulness practices.

### 4.2 Effects on humanistic care competence

Humanistic care competence was higher across groups, with the strongest associations in the combined intervention group. Scores continued to trend higher at follow-up, indicating sustained associations. These patterns support prior evidence that reflective and mindfulness-based approaches are linked to greater caring behaviors and professional competence (2).

The associations between empathy and humanistic care competence may partly explain these findings, as empathy has been shown to partially mediate the relationship between professional competence and caring ability (3). Additionally, emotional intelligence may influence resilience, and higher empathy may contribute to stronger humanistic care, suggesting potential interrelationships among the outcomes. While these relationships were not directly tested in this study, they are supported by prior literature and provide a conceptual framework for understanding how these psychological and professional competencies may interact (17). The differences observed between groups may reflect the additive benefit of mindfulness, with the combined intervention supporting both intrapersonal and interpersonal capacities more effectively than Balint alone. Furthermore, cultural expectations in China regarding patient-centered care and professional diligence may have shaped nurses’ engagement with humanistic care practices.

### 4.3 Effects on emotional intelligence

Emotional intelligence was higher in the combined intervention group and continued to trend higher at follow-up. Mindfulness-based approaches are associated with better emotional awareness and regulation, consistent with the mindfulness emotional regulation model, which suggests reductions in automatic emotional responses and improved adaptive processing (9, 11, 18).

The Balint component may also support these associations by helping nurses articulate complex emotional experiences during case discussions. Together, these approaches provide conceptual understanding and practical strategies linked to better emotional regulation, such as RAIN (Recognize, Allow, Investigate, Nurture) and emotional awareness exercises, which may help nurses manage difficult emotions in clinical contexts. Emotional intelligence is closely associated with nursing competence and humanistic care, reinforcing the relevance of these combined interventions (6, 17). The modest improvements in the Balint-only group highlight the importance of reflective discussion in cultivating emotional awareness, though the combined approach appears to offer broader enhancement. Cultural factors emphasizing self-control and emotional moderation may have facilitated the integration of these strategies into professional practice.

### 4.4 Effects on psychological resilience

Psychological resilience was higher across all groups, with the strongest associations observed in the combined intervention group. This finding aligns with prior research showing that mindfulness-based interventions are associated with greater resilience and lower stress among healthcare professionals (19–21). A systematic review by Burton et al. found that MBSR was associated with improvements in psychological outcomes, including resilience, stress, and burnout (19).

The higher resilience scores may reflect associations with increased acceptance, cognitive flexibility, and adaptive coping strategies through mindfulness practice. Mindfulness-based approaches are linked to enhanced emotion regulation and cognitive flexibility, which may support more balanced and resilient responses to stress (18, 21). Resilience is also associated with maintaining professional performance and reducing burnout risk among nurses (22). Taken together, these findings suggest a possible network of interrelated outcomes, where improvements in emotional intelligence may reinforce resilience, which in turn may enhance professional care behaviors, although further empirical testing is needed. Notably, the Balint-only group showed smaller gains in resilience, indicating that while reflective discussion may support coping skills, mindfulness may be essential for substantial improvements in adaptive stress responses. Cultural norms promoting endurance, collective responsibility, and coping with challenges may have contributed to the resilience outcomes observed in this study.

### 4.5 Mechanisms of the combined intervention

The combined intervention is associated with higher outcomes potentially due to dual mechanisms; however, it is important to note that these mechanisms are theoretical and were not empirically tested in this study. Balint groups are primarily linked to improved interpersonal competence by enhancing understanding of nurse–patient relationships and promoting reflective practice, whereas MBSR is associated with improvements in intrapersonal processes, such as emotional regulation, stress management, and self-awareness. The integration of these approaches may theoretically support simultaneous associations with both relational skills and internal psychological resources. This conceptual framework aligns with existing models of nursing competence, emphasizing interactions among cognitive, emotional, and behavioral domains (23). While these mechanisms provide plausible explanations for the observed improvements in empathy, emotional intelligence, and resilience, no mediation or pathway analyses were conducted to confirm these relationships. Future research should empirically examine these potential pathways using statistical modeling, such as mediation or structural equation analyses, to validate the theoretical interpretations presented here. Cultural factors should also be considered in future studies, as variations in cultural values and professional norms may influence the mechanisms through which these interventions operate.

### 4.6 Comparison with alternative interventions

While the present study focused on Balint + MBSR, other stress management and professional development interventions exist, including cognitive-behavioral programs, simulation-based training, and peer-support initiatives. Evidence suggests that cognitive-behavioral interventions may improve emotional regulation and resilience, though they often focus primarily on intrapersonal coping rather than interpersonal skills (24, 25). Simulation−based education has been demonstrated to enhance communication competence, self−efficacy, and relational skills in nursing students and practicing nurses, supporting clinical communication and professional confidence (26, 27). In comparison, the integration of Balint groups with MBSR provides a dual approach that targets both interpersonal understanding and intrapersonal awareness, potentially offering broader benefits across empathy, emotional intelligence, and resilience. Recent studies (2023–2025) further support the effectiveness of mindfulness and reflective interventions in strengthening emotional intelligence and resilience mechanisms in nursing populations, indicating that combined approaches may be superior to single-method interventions in promoting holistic professional competence (10, 28).

### 4.7 Feasibility and practical implications

The intervention was implemented without major issues and was generally well-received by participating nurses. However, it is important to note that adherence, dropout, and participant satisfaction were not systematically measured in this study, limiting the strength of claims regarding feasibility and acceptability. The structure of weekly sessions lasting 1.5–2 hours over 8 weeks was compatible with the demanding schedules of obstetric nurses. Previous studies have also shown that MBSR programs are feasible and linked to improved well-being and reduced burnout among nurses (29, 30).

From a practical perspective, integrating Balint group activities with mindfulness training may be associated with better psychological well-being and professional competence. Such interventions could be incorporated into continuing education programs or workplace mental health initiatives. Attention to cultural factors is critical when implementing similar interventions in different contexts, as responsiveness to reflective practices and mindfulness may vary according to local norms and values. Future studies should systematically collect adherence, dropout, and satisfaction data to more rigorously evaluate feasibility.

### 4.8 Limitations and future directions

Several limitations should be considered. First, this study was conducted in a single center with a relatively small sample size, which may limit generalizability. Second, the follow-up period was limited to 6 weeks, and long-term effects remain unclear. Third, all outcomes were assessed using self-report measures, which may introduce response bias. Fourth, the study lacked blinding, which could have introduced performance bias. Fifth, intervention fidelity was not systematically measured, limiting the ability to verify whether the intervention was delivered consistently across sessions. Additionally, potential confounding factors such as workload, shift patterns, and prior experience with stress-management techniques were not controlled, which may have influenced the observed outcomes. Barriers encountered during implementation were not formally recorded, reducing insight into practical challenges of the intervention. Effect sizes were not consistently reported, and the study did not include a formal power analysis to guide the interpretation of nonsignificant results. Furthermore, some mediators in the original analysis conceptually overlapped with the outcome measures, which could lead to tautological associations. These items were removed in the revised analysis to ensure that mediators were conceptually distinct from the outcome, strengthening the validity of the observed associations. The handling of missing values has also been revised. Instead of using linear interpolation, which is inappropriate for cross-sectional data, missing values in control variables were addressed using multiple imputation, which is suitable for single-wave surveys and reduces potential bias. The use of wives’ weekly exercise frequency as an instrumental variable (IV) for wives’ depression has important limitations. The exclusion restriction is unlikely to hold because wives’ exercise could plausibly affect husbands’ well-being through multiple channels beyond depression, including shared activities, household routines, social participation, and husbands’ own exercise. Our mechanism analysis further suggests that husbands’ exercise may mediate these associations, making causal interpretation less credible. Accordingly, results from the IV analysis are presented as associations rather than causal effects. Despite these limitations, the study has several strengths. It employed a randomized controlled design with a three-arm structure, enabling comparison between a combined intervention, a single intervention, and routine practice. Interventions were carefully developed based on prior evidence and adapted to the clinical characteristics of obstetric nursing, ensuring feasibility and relevance. Multiple assessment time points allowed examination of sustained associations, and validated instruments were used to measure outcomes.

The findings have several practical implications. Integrating Balint group and mindfulness-based stress reduction interventions may be associated with improved empathy, humanistic care competence, emotional intelligence, and resilience among nurses. In clinical practice, these interventions could be incorporated into workplace programs to enhance nurses’ professional quality and well-being. In nursing education, reflective and mindfulness-based components could be included in training curricula to strengthen both interpersonal and intrapersonal competencies. For healthcare policy, the results support the development of interventions and continuing education programs aimed at reducing stress and promoting professional well-being in high-demand clinical environments.

Future research should address the limitations highlighted above by including multicenter designs, larger sample sizes, longer follow-up periods, objective outcome measures, blinding where feasible, systematic monitoring of intervention fidelity, and careful control of confounders. Qualitative studies may provide deeper insights into participants’ experiences, and objective measures such as physiological or neurobiological indicators could be used to explore underlying mechanisms. Further investigation is also needed to examine individual differences in intervention response, such as personality traits and prior experience. Future studies could also explore alternative instruments, placebo tests, falsification exercises, or balance checks to strengthen causal inference. Additionally, future trials should report effect sizes and conduct power analyses to support more rigorous interpretation of results and guide sample size calculations.

## 5 Conclusion

In conclusion, the combined Balint group and mindfulness-based stress reduction intervention was associated with higher empathy, humanistic care competence, emotional intelligence, and psychological resilience among obstetric nurses, with stronger associations than those observed for the Balint group alone. The intervention is feasible, acceptable, and linked to improvements in professional quality and psychological well-being, with important implications for nursing practice and patient care.

## Data Availability

The raw data supporting the conclusions of this article will be made available by the authors, without undue reservation.
